# Left Atrial Enlargement and the Risk of Stroke: A Meta-Analysis of Prospective Cohort Studies

**DOI:** 10.3389/fneur.2020.00026

**Published:** 2020-02-14

**Authors:** Yicheng Xu, Liming Zhao, Lvming Zhang, Yalei Han, Peifu Wang, Shengyuan Yu

**Affiliations:** ^1^Department of Neurology, Aerospace Center Hospital, Beijing, China; ^2^Department of Neurology, Changyi People's Hospital, Weifang, China; ^3^Department of Neurology, Chinese People's Liberation Army General Hospital, Beijing, China

**Keywords:** left atrial size, stroke, meta-analysis, risk, systematic review

## Abstract

**Background:** The association between left atrial size and the risk of stroke has not been fully understood. We performed a meta-analysis of prospective cohort studies to determine whether left atrial enlargement (LAE) is associated with an increased risk of stroke.

**Methods:** We searched PubMed, EMBASE, Web of Science, and the Cochrane Library through May 2019. Prospective cohort studies were included if they reported hazard ratios (HRs) and corresponding 95% confidence intervals (CIs) of stroke with respect to LAE. All meta-analyses were performed using a random-effects model.

**Results:** Six studies involving 66,007 participants and 3,549 stroke events were included. Compared with patients without LAE, those with LAE had an increased risk of stroke (adjusted HR 1.68, 95% CI 1.36–2.07). There was also a graded association with stroke relating to LAE (adjusted HR for mild LAE 1.50, 95% CI 0.98–2.28; moderate LAE 1.40, 95% CI 1.12–1.75; and severe LAE 1.59, 95% CI 1.33–1.90). Furthermore, for each increase of 1 cm in left atrial diameter, the odds of stroke were increased by 24% (adjusted HR 1.24, 95% CI 1.03–1.50).

**Conclusions:** Our meta-analysis demonstrated that LAE is associated with an increased and graded risk of stroke.

## Introduction

Stroke is a catastrophic condition with a great burden of disability and death. Furthermore, as the world population is aging, the global burden of stroke will increase dramatically (1). And it has been predicted that there will be almost 12 million stroke deaths and 70 million stroke survivors by 2030 (2). Thus, to identify the high risks of stroke patients and to manage well stroke risk factors have an important individual and social impact.

Studies have found that left atrial size was associated with an increased risk of ischemic stroke and all-cause of deaths despite of controversy (3–5). Additionally, some studies have found that this association between left atrial size and stroke seems to exist independent of atrial fibrillation (AF) (6, 7). Therefore, it is important to identify the association between left atrial size and stroke, which would be helpful to stratify the risk of stroke.

Among various quantification methods of left atrial size, the European Society of Cardiology recommends that left atrial volume indexed to body surface area (LAVi) should be used to predict all-cause mortality and ischemic stroke because of its accuracy and reliability (8), considering that it is simpler, more widely available, and more widely used in clinical practice. Most of studies chose left atrial diameter (LAD) as their measurement. Hereby, we conducted a systematic review and meta-analysis of all the prospective cohort studies to determine the association between LAD and the risk of stroke.

## Methods

This meta-analysis was conducted and reported according to the Preferred Reporting Items for Systematic Reviews and Meta-Analyses statement (9). It was prospectively registered in the PROSPERO registry (CRD42017074455).

### Data Sources and Search Strategy

We searched PubMed (from inception to May 2019), EMBASE (from inception to May 2019), Web of Science, and the Cochrane Library Databases (May 2019). The following search terms were used: “left atrial volume” OR “left atrial size” OR “left atrial dimension” OR “left atrial diameter” OR “left atrial dilatation” OR “left atrial hypertrophy” OR “left atrial enlargement” OR “left atria volume” OR “left atria size” OR “left atria dimension” OR “left atria diameter” OR “left atria dilatation” OR “left atria hypertrophy” OR “left atria enlargement” OR “left atrium volume” OR “left atrium size” OR “left atrium dimension” OR “left atrium diameter” OR “left atrium dilatation” OR “left atrium hypertrophy” OR “left atrium enlargement” OR “enlarged left atrium” OR “enlarged left atria” OR “heart atrium enlargement” OR atriomega^*^ AND “cerebral embolism” OR stroke OR “Apoplexy” OR “Brain Vascular Accident” OR CVA OR “Cerebrovascular Accident^*^” OR “ischemic infarction” OR embolism^*^ OR thromboembolism^*^ OR thrombosis. The Medical Subject Headings (MeSH) search and free-text terms search were conducted with our search strategy. Furthermore, we hand-reviewed reference lists of included studies and review articles to search for more studies. Only those that were published as full-length articles and in English were considered. Two investigators (YCX and LMZ) independently performed the literature search.

### Study Selection and Eligibility Criteria

We removed duplicates, screened the titles and abstracts for relevance, and accessed full text to identify eligibility. For inclusion, studies had to fulfill the following criteria: have a prospective cohort design with follow-up >1 year; report relative risks or hazard ratios (HRs) and their corresponding 95% confidence intervals (CIs) of stroke relating to left atrial enlargement (LAE).

### Data Extraction

Two authors (YCX and LMZ) independently collected data from all included studies using a standard form. The following information was extracted from each included study: first author, year of publication, country of the study, participant number and characteristics, sex, AF status at baseline, exposure definition, mean length of follow-up, number of stroke events, and covariates in the fully adjusted model.

### Quality Assessment

We assessed the quality of each cohort by using the Newcastle–Ottawa Scale (10). This scale consists of three domains: selection, comparability, and outcome. The maximum score for cohort is 9 points. We awarded points of 0 to 3, 4 to 6, and 7 to 9 for low, moderate, and high quality, respectively.

### Summary Measures

We described associations using HR or risk ratio (RR) for prospective studies. RR was considered equivalent to HR (11).

### Statistical Analysis

We pooled the adjusted HRs and corresponding 95% CIs from each study using a random-effects model. We assessed heterogeneity across studies using the *Q* statistic and the *I*^2^ statistic. The *I*^2^ statistic is used to estimate the proportion of total variation across studies due to heterogeneity rather than chance (ranging from 0 to 100%). An *I*^2^ value > 50% indicates significant heterogeneity. We also performed subgroup analyses according to LA size to further determine the relationship between LAE and the risk of stroke. To evaluate the publication bias, we constructed a funnel plot to visualize possible asymmetry (12). All statistical analyses were performed with Stata, version 12 (StataCorp, College Station, TX). A *P* < 0.05 was considered statistically significant.

## Result

### Literature Search

**Figure 1:**
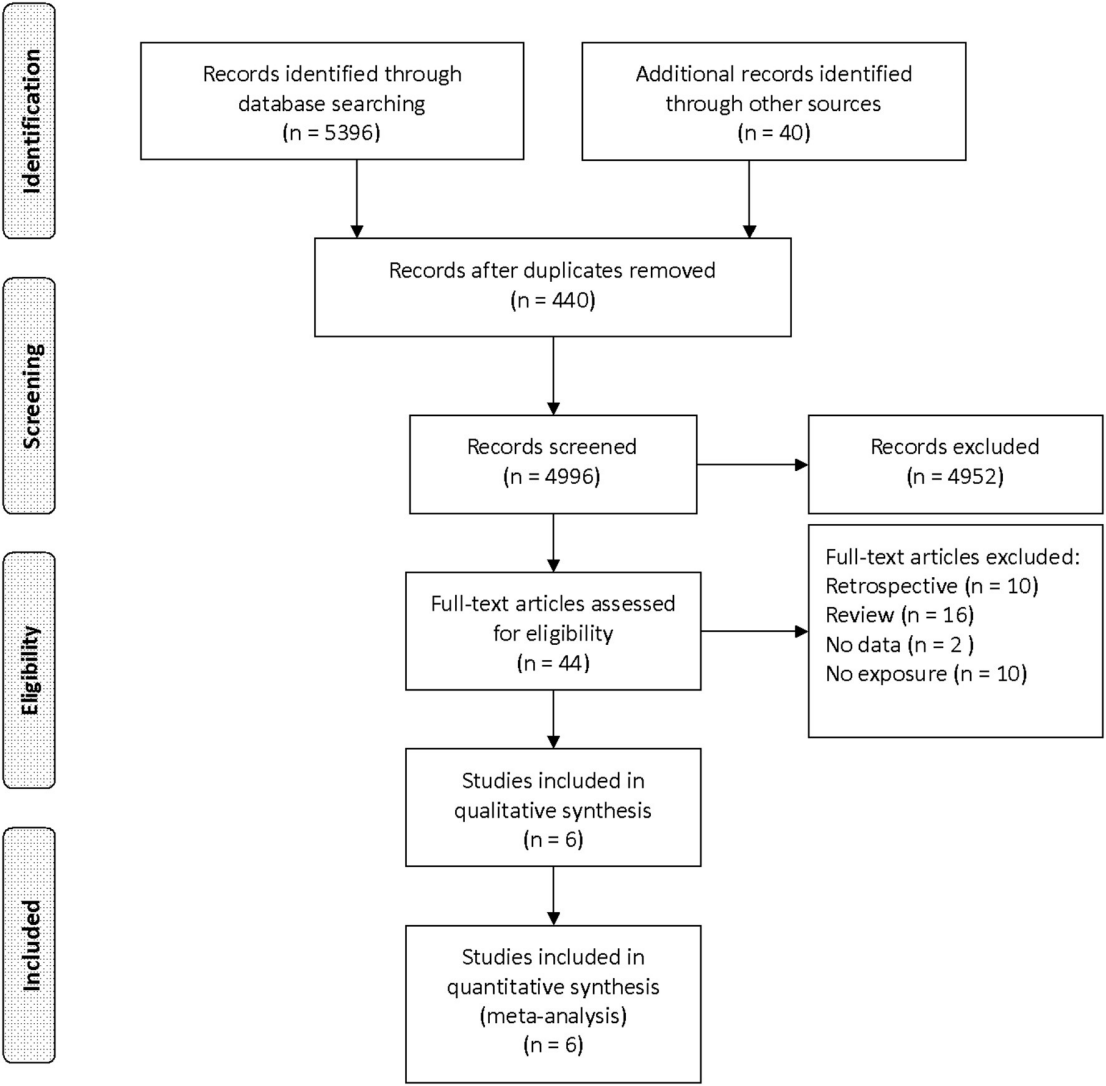
Preferred reporting items for systematic reviews and meta-analyses (PRISMA) flow diagram.

Our initial search yielded 5,436 records. After screening titles and abstracts, we obtained 44 articles for full-text reviewing. Finally, six studies were eligible for inclusion (Figure 1) (7, 13–17).

### Study Characteristics

The characteristics of the included studies are shown in Table 1. These studies were published between 1995 and 2016. The sample sizes of the cohorts ranged from 593 to 52,639. The mean age of the participants varied from 51 to 73.6 years. Among these six studies, only two studies (14, 15) excluded AF patients from baseline participants. The lengths of follow-up for incident stroke varied between 2.6 and 13 years. All the studies reported the association between LAE and stroke after fully adjusting for traditional cardiovascular risk factors such as age, sex, hypertension, and diabetes. The meta-analysis consisted of 66,007 participants and 3,549 stroke events. The baseline participant characteristics among included studies are shown in Table 2. Quality assessment of each study is outlined in Table 3. The score for all studies is 7 or above with high quality.

**Table 1:** Characteristics of six prospective studies contributing data to the current analysis.

**Study**	**Population (number)** **(% men)**	**Definition of LAE**	**LAD category**	**End points**	**No. of stroke events**	**HR** **(95% CI)**	**Covariates in the fully adjusted model**
Benjamin et al. (17), USA	General population 55 years and older (*n* = 3,099) (44%)	No report	Categorical (Tertile 1, Tertile 2, Tertile 3)	Stroke	137	Men: 2.4 (1.6–3.7)/1 cmWomen: 1.4 (0.9–2.1)/1 cm	Adjusted for age, hypertension, diabetes, ECG left ventricular hypertrophy, smoking, prevalent atrial fibrillation, and prevalent congestive heart failure or myocardial infarction
Bouzas-Mosquera et al. (13), Spain	Patients who underwent echocardiography (*n* = 52,639) (52.9%)	Men: LAD ≥ 4.1 cm Women: LAD ≥ 3.9 cm	Categorical (normal, mild, moderate, severe)	Ischemic stroke,	2,314	1.36 (1.23–1.51)	Adjusted for age, sex, hypertension, diabetes, hypercholesterolemia, history of smoking, previous stroke or transient ischemic attack, history of atrial fibrillation, severity of mitral regurgitation, mitral valve stenosis, history of mitral valve intervention, history of congestive heart failure, prior myocardial infarction, history of coronary revascularization, left ventricular ejection fraction, end-diastolic left ventricular diameter, left ventricular mass, history of cancer, chronic kidney disease, chronic obstructive pulmonary disease, anticoagulant therapy, referral setting
Karas et al. (14), USA	General population (*n* = 2,391) (35.4%)	Men: LAD > 4.2 cm Women: LAD > 3.8 cm	Continuous	Ischemic stroke	138	1.99 (1.28–3.07)	Adjusted for age, sex, body mass index, systolic blood pressure, antihypertensive therapy, diabetes, low-density lipoprotein, high-density lipoprotein, current smoking, serum creatinine, UACR, and HbA1c
Hamatani et al. (7), Japan	Patients with non-valvular AF (*n* = 2,713) (60.4%)	LAD > 45 mm	Categorical (LAE and non-LAE)	Stroke and SE	Ischemic stroke 112; hemorrhagic stroke 43; SE 4	1.74 (1.25–2.42)	Adjusted for CHA2DS2-VASc score, OAC prescription and age
Haruki et al. (15), Japan	patients with HCM without AF (*n* = 593) (62.9%)	LAD ≥ 48 mm	Categorical (LAE and non-LAE) (LAD ≥48 mm)	Stroke and SE	Ischemic stroke 62; hemorrhagic stroke 4; SE 6	2.74 (1.2–6.23)	Adjusted for age, sex, family history of sudden cardiac death, unexplained syncope, left ventricular intracavitary gradient, maximum left ventricular wall thickness ≥ 30 mm, non-sustained ventricular tachycardia
Broughton et al. (16), USA	Population 65 years and older (*n* = 4,572) (43%)	Men: LAD ≥ 4.1 cm Women: LAD ≥ 3.9 cm	Categorical (normal, mild, moderate, severe)	Ischemic stroke	739	1.81 (1.4–2.34)	Adjusted for age, sex, race, education, income, smoking, diabetes, body mass index, low-density lipoprotein cholesterol, systolic blood pressure, antihypertensive medications, aspirin, heart failure, and peripheral arterial disease.

*LAE, left atrial enlargement; LAD, left atrial diameter; HCM: hypertrophic cardiomyopathy; SE, systemic embolism; HR, hazard ratio; CI, confidence interval; UACR, urinary albumin-to-creatinine ratio; HbA1c, glycated hemoglobin; AF, atrial fibrillation; OAC, oral anticoagulant*.

**Table 2:** Baseline participant characteristics among included studies.

**Study**	**Mean age, years**	**Hypertension** **(%)**	**Diabetes** **(%)**	**Smoking** **(%)**	**Previous** **stroke or TIA** **(%)**	**AF** **(%)**	**CHADS2 score** **(mean)**	**Follow-up, years**
Benjamin et al. (17), USA	64.1	38.1	8	24.5	0	1.8	No report	8
Bouzas-Mosquera et al. (13), Spain	61.8	48.3	25.4	24	9.3	21.4	No report	5.5
Karas et al. (14), USA	59.1	No report	45.3	31.3	0	0	No report	12
Hamatani et al. (7), Japan	73.6	62.2	23.6	35	21	100	2.37	2.6
Haruki et al. (15), Japan	51	No report	No report	No report	No report	0	No report	10.7
Broughton et al. (16), USA	>65	No report	14.4	53.3	0	5	No report	13

*TIA, transient ischemic attack; AF, atrial fibrillation*.

**Table 3:** Quality ratings for the six cohort studies included on the basis of Newcastle-Ottawa quality assessment scale.

	**Selection (score)**				**Comparability (score)**	**Outcome (score)**			**Total score**
	**Representative of exposed cohort**	**Selections of non-exposed cohort**	**Assessment of exposure**	**Absence of outcome at start of study**	**Control for age or hypertension or DM or smoking**	**Assessment of outcome**	**Follow-up period**	**Adequacy of follow-up**	
Benjamin et al. (17)	1	1	1	1	2	1	1	1	9 (high)
Bouzas-Mosquera et al. (13)	1	1	1	0	2	1	1	1	8 (high)
Karas et al. (14)	1	1	1	0	2	1	1	1	8 (high)
Hamatani et al. (7)	1	1	1	0	2	1	1	0	7 (high)
Haruki et al. (15)	1	1	1	1	1	1	1	1	8 (high)
Broughton et al. (16)	1	1	1	1	2	1	1	1	9 (high)
								Mean	8.2

*DM, diabetes mellitus*.

### Left Atrial Enlargement and Stroke

**Figure 2:**
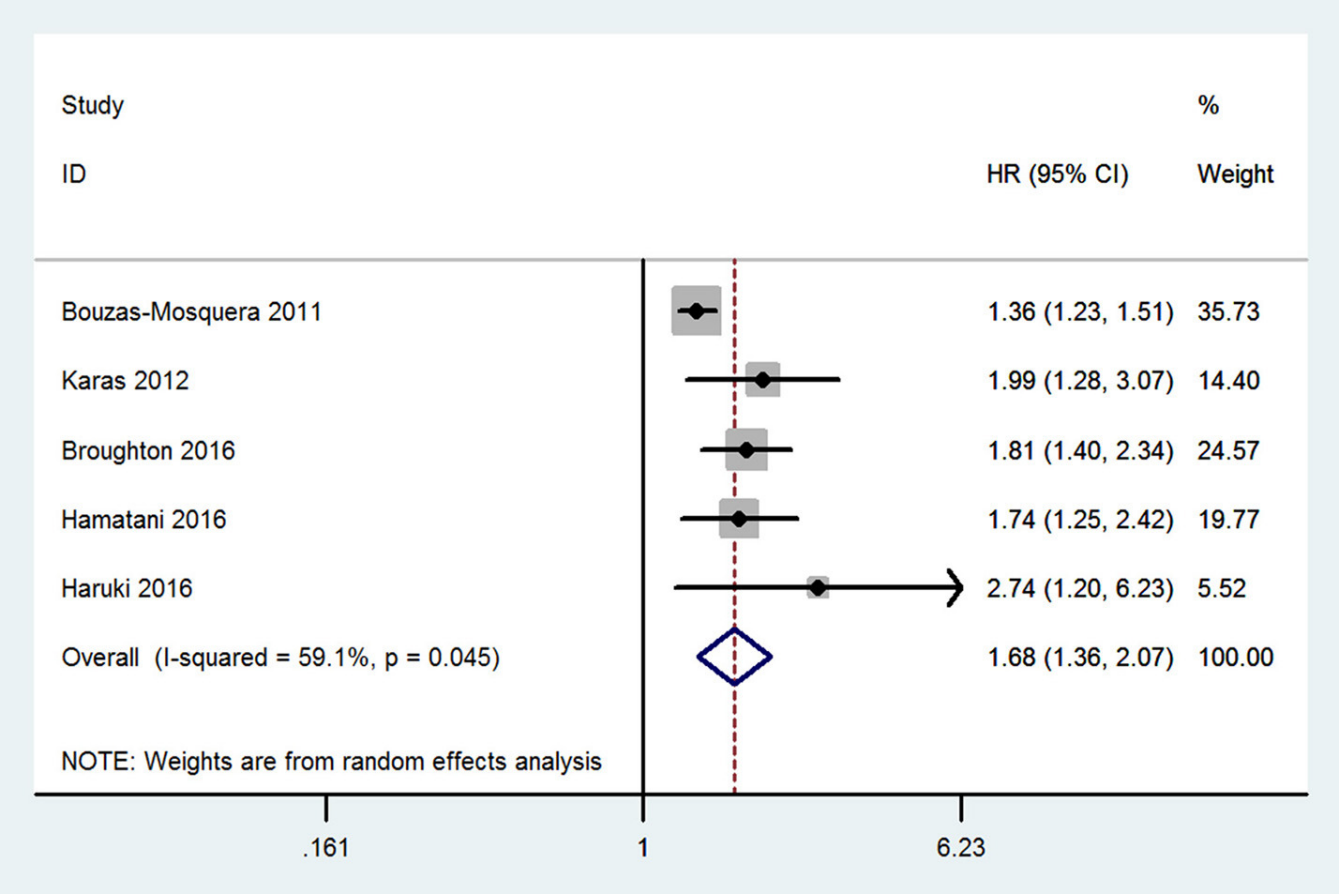
Meta-analyses of hazard ratios for the association between left atrial enlargement and stroke.

**Figure 3:**
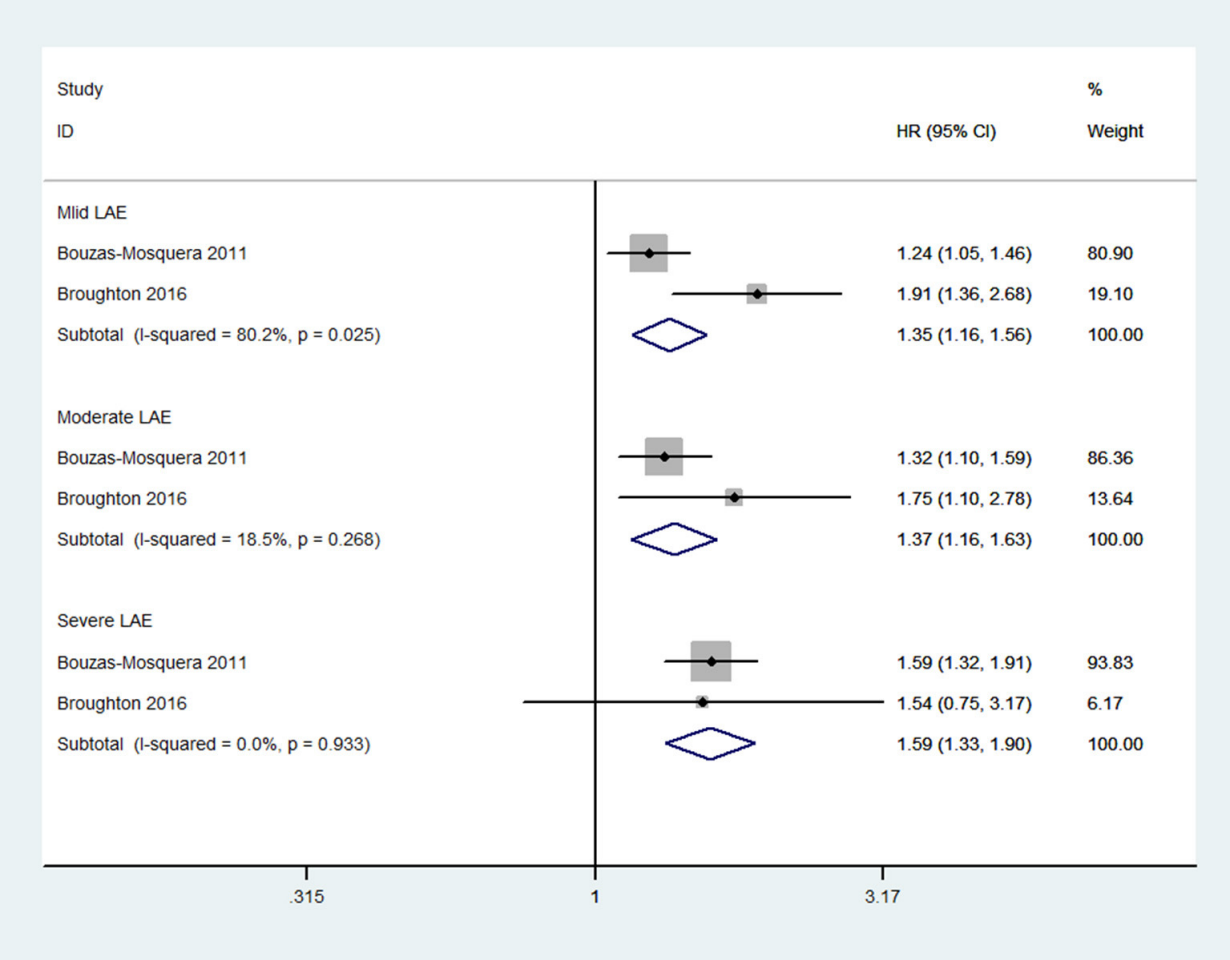
Meta-analyses of hazard ratios for the association between the severity of left atrial enlargement and stroke.

**Figure 4:**
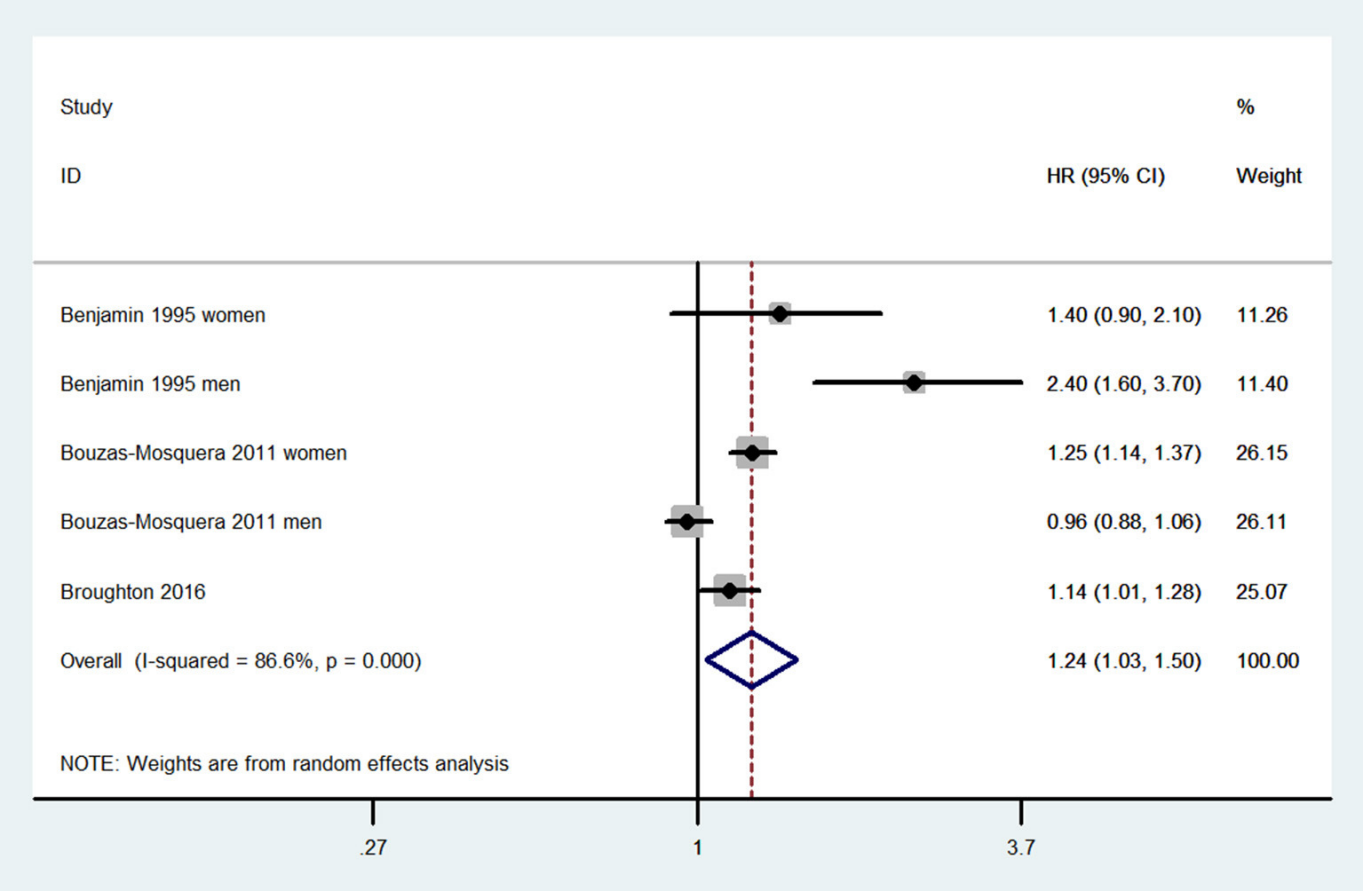
Meta-analyses of hazard ratios for the association between continuous left atrial diameter and stroke.

**Figure 5:**
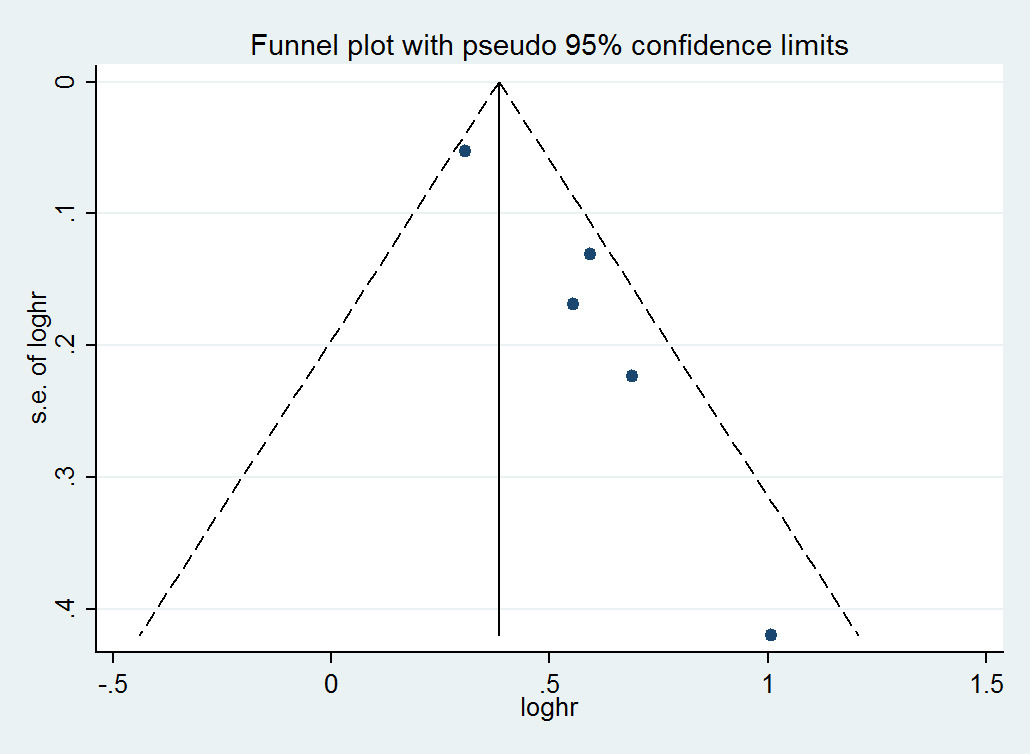
Funnel plot with pseudo 95% confidence limits.

Compared with patients without LAE, those with LAE had an increased risk of stroke (five studies; adjusted HR 1.68, 95% CI 1.36–2.07; Figure 2). Figure 3 shows the association between the LAE grades and risk of stroke. The pooled HR was 1.50 for mild LAE (95% CI 0.98–2.28; two studies), 1.40 for moderate LAE (95% CI 1.12–1.75; two studies), and 1.59 for severe LAE (95% CI 1.33–1.90; two studies). Furthermore, for each increase of 1 cm in LAD, the odds of stroke were increased by 24% (adjusted HR 1.24, 95% CI 1.03–1.50; Figure 4). Visual inspection of the funnel plot showed some asymmetry (Figure 5). Considering that only five studies were included in this meta-analysis, it was hard for us to rule out the publication bias.

## Discussion

Our meta-analysis of five prospective cohort studies indicates that LAE is associated with increased risk of stroke. Additionally, we found that the risk of stroke became higher with the increase of the LAD. For each increase of 1 cm in LAD, the odds of stroke were increased by 24%.

The mechanisms related LAE to stroke have not been fully understood. Some hypotheses have been proposed, which might partially explain the association between LAE and stroke. First, LAE may predispose the development of AF (18), which is a well-established risk factor for stroke. Second, LAE was considered as a surrogate marker of other risk factors for cardiovascular events (19, 20) such as hypertension or carotid atherosclerosis, which are significantly associated with the high risk of stroke. Third, LAE may promote blood stasis, which results in the increased risk of potential embolization through induction of thrombus formation (7). Additionally, this theory has been supported by the studies of transesophageal echocardiography (TEE) that LAE was associated with spontaneous echocardiographic contrast and LA thrombus (21).

The investigation of the association between LAE and stroke may have several clinical implications. First, much evidence has been found that the left atrial structure and function were strongly associated with the risk of new-onset AF (22, 23) and intermittent AF (24). Meanwhile, the relationship between the AF (including intermittent AF) and ischemic stroke (which was also includes cryptogenic ischemic stroke) is well-recognized (25, 26). So current techniques for detection AF of stroke patients are expensive and have low yield (27). And studies have found that the measurement of left atrial function could predict AF in stroke patients (28). It may be proposed that the left atrial size could serve as a more ideal and stable predictor than AF/underlying AF in ischemic stroke patients, especially in cryptogenic ischemic stroke, which would be also helpful in differentiating etiological diagnoses of ischemic stroke and treatment of the ischemic stroke: choosing or not choosing oral anticoagulant (OAC) treatment. Second, a study (29) that investigated the relationship between LAE and ischemic stroke had found on TTE that LAE had a satisfying predictive value for left atrial thrombus (LAT) on TEE. Thus, patients will not need to undergo TEE, which is expensive and invasive and has a higher risk of complications. Third, based on the above findings and implications, LAD, as the simplest measurement, can be proposed as an additional risk stratification refinement for patients in predicting ischemic stroke and systemic embolism, (7) which is also helpful in the identification of high-risk patients who could benefit from preventive OAC treatment. Fourth, LAE was considered as a surrogate marker of other risk factors for cardiovascular events. So it may be advocated that physicians can optimize risk factor management through monitoring the change of LAE (30).

Several limitations of this study should be considered. First, the differences of population in the included studies could result in inconsistency between studies. And it might be a significant limitation of our study. However, we only included prospective cohort studies, which helped minimize heterogeneity. Second, as we only included studies published in English, we might neglect studies published in other languages and produce bias. Third, the methodological quality of the selected studies was not uniform. Fourth, we considered that RR was equivalent to HR owing to the small number of the included studies in the present meta-analysis. So the findings of our study should be interpreted cautiously.

## Conclusions

In conclusion, our meta-analysis demonstrated that LAE is associated with an increased and graded risk of stroke. Future studies should explore the risk and benefit of thromboprophylaxis in general populations with LAE.

## Data Availability Statement

The authors confirm that all data underlying the findings are fully available without restriction. All relevant data are within the article.

## Author Contributions

YX conceived the study, participated in the design, collected the data, performed statistical analyses, and drafted the manuscript. LiZ participated in the design, collected the data, performed statistical analyses, and drafted the manuscript. LvZ and YH performed statistical analyses and helped to draft the manuscript. PW and SY performed statistical analyses and helped to revise the manuscript critically for important intellectual content. All authors read and approved the final manuscript.

### Conflict of Interest

The authors declare that the research was conducted in the absence of any commercial or financial relationships that could be construed as a potential conflict of interest.

## Abbreviations

AF: atrial fibrillation
CIs: confidence intervals
HR: hazard ratio
LAD: left atrial diameter
LAVi: left atrial volume indexed to body surface area
MeSH: Medical Subject Headings
OAC: oral anticoagulant
RR: risk ratio
TTE: transesophageal echocardiography.

## References

[1] Bejot Y Daubail B Giroud M. Epidemiology of stroke and transient ischemic attacks: current knowledge and perspectives. Revue Neurol. (2016) 172:59–68. 10.1016/j.neurol.2015.07.013 26718592

[2] Feigin VL Forouzanfar MH Krishnamurthi R Mensah GA Connor M Bennett DA. Global and regional burden of stroke during 1990-2010: findings from the Global Burden of Disease Study 2010. Lancet. (2014) 383:245–54. 10.1016/S0140-6736(13)61953-4 24449944 PMC4181600

[3] Aziz EF Kukin M Javed F Musat D Nader A Pratap B. Right ventricular dysfunction is a strong predictor of developing atrial fibrillation in acutely decompensated heart failure patients, ACAP-HF data analysis. J Card Fail. (2010) 16:827–34. 10.1016/j.cardfail.2010.05.004 20932465

[4] Biteker M Kayatas K Basaran O Dogan V Ozlek E Ozlek B. The role of left atrial volume index in patients with a first-ever acute ischemic stroke. J Stroke Cerebrovasc Dis. (2017) 26:321–6. 10.1016/j.jstrokecerebrovasdis.2016.09.023 27773589

[5] Gardner JD Skelton WPt Khouzam RN. Is it time to incorporate the left atrial size to the current stroke risk scoring systems for atrial fibrillation? Curr Prob Cardiol. (2016) 41:251–9. 10.1016/j.cpcardiol.2016.10.004 27899169

[6] Barnes ME Miyasaka Y Seward JB Gersh BJ Rosales AG Bailey KR. Left atrial volume in the prediction of first ischemic stroke in an elderly cohort without atrial fibrillation. Mayo Clin Proc. (2004) 79:1008–14. 10.4065/79.8.1008 15301328

[7] Hamatani Y Ogawa H Takabayashi K Yamashita Y Takagi D Esato M. Left atrial enlargement is an independent predictor of stroke and systemic embolism in patients with non-valvular atrial fibrillation. Sci Rep. (2016) 6:31042. 10.1038/srep31042 27485817 PMC4971566

[8] Hypertension EETFftMoA 2013 Practice guidelines for the management of arterial hypertension of the European Society of Hypertension (ESH) and the European Society of Cardiology (ESC): ESH/ESC Task Force for the Management of Arterial Hypertension. J Hypertens. (2013) 31:1925–38. 10.1097/HJH.0b013e328364ca4c 24107724

[9] Moher D Liberati A Tetzlaff J Altman DG Group P. Preferred reporting items for systematic reviews and meta-analyses: the PRISMA statement. BMJ. (2009) 339:b2535. 10.1136/bmj.b2535 19622551 PMC2714657

[10] Wells GA Shea B O'Connell D Peterson J Welch V Losos M The Newcastle-Ottawa Scale (NOS) for Assessing the Quality of Nonrandomised Studies in Meta-Analyses. Available online at: http://www.ohri.ca/programs/clinical_epidemiology/oxford.asp (accessed February 5, 2020).

[11] Wang D Li W Cui X Meng Y Zhou M Xiao L. Sleep duration and risk of coronary heart disease: A systematic review and meta-analysis of prospective cohort studies. Int J Cardiol. (2016) 219:231–9. 10.1016/j.ijcard.2016.06.027 27336192

[12] Sterne J Egger M. Funnel plots for detecting bias in meta-analysis: guidelines on choice of axis. J Clin Epidemiol. (2001)54: 1046–55. 10.1016/S0895-4356(01)00377-8 11576817

[13] Bouzas-Mosquera A Broullon FJ Alvarez-Garcia N Méndez E Peteiro J Gándara-Sambade T. Left atrial size and risk for all-cause mortality and ischemic stroke. CMAJ. (2011) 183:E657–64. 10.1503/cmaj.091688 21609990 PMC3134756

[14] Karas MG Devereux RB Wiebers DO Whisnant JP Best LG Lee ET. Incremental value of biochemical and echocardiographic measures in prediction of ischemic stroke: the Strong Heart Study. Stroke. (2012) 43:720–6. 10.1161/STROKEAHA.111.631168 22207511 PMC3288714

[15] Haruki S Minami Y Hagiwara N. Stroke and embolic events in hypertrophic cardiomyopathy: risk stratification in patients without atrial fibrillation. Stroke. (2016) 47:936–42. 10.1161/STROKEAHA.115.012130 26941260

[16] Broughton ST O'Neal WT Salahuddin T Soliman EZ. The influence of left atrial enlargement on the relationship between atrial fibrillation and stroke. J Stroke Cerebrovasc Dis. (2016) 25:1396–402. 10.1016/j.jstrokecerebrovasdis.2016.02.004 27012217

[17] Benjamin EJ D'Agostino RB Belanger AJ Wolf PA Levy D. Left atrial size and the risk of stroke and death. The Framingham Heart Study. Circulation. (1995) 92:835–41. 10.1161/01.CIR.92.4.835 7641364

[18] Vaziri SM Larson MG Benjamin EJ Levy D. Echocardiographic predictors of nonrheumatic atrial fibrillation. The Framingham Heart Study. Circulation. (1994) 89:724–30. 10.1161/01.CIR.89.2.724 8313561

[19] Nagarajarao HS Penman AD Taylor HA Mosley TH Butler K Skelton TN. The predictive value of left atrial size for incident ischemic stroke and all-cause mortality in African Americans: the Atherosclerosis Risk in Communities (ARIC) Study. Stroke. (2008) 39:2701–6. 10.1161/STROKEAHA.108.515221 18658033 PMC3292848

[20] Pierdomenico SD Pierdomenico AM Di Carlo S Di Tommaso R Cuccurullo F. Left atrial enlargement and risk of ischemic stroke in elderly treated hypertensive patients. Am J Hypertens. (2014) 27:1179–84. 10.1093/ajh/hpu042 24682334

[21] Providencia R Botelho A Trigo J Quintal N Nascimento J Mota P. Possible refinement of clinical thromboembolism assessment in patients with atrial fibrillation using echocardiographic parameters. Europace. (2012) 14:36–45. 10.1093/europace/eur272 21868410

[22] Psaty BM Manolio TA Kuller LH Kronmal RA Cushman M Fried LP. Incidence of and risk factors for atrial fibrillation in older adults. Circulation. (1997) 96:2455–61. 10.1161/01.CIR.96.7.2455 9337224

[23] Patel DA Lavie CJ Milani RV Shah S Gilliland Y. Clinical implications of left atrial enlargement: a review. Ochsner J. (2009) 9:191–6. 21603443 PMC3096293

[24] Kinay O Nazli C Ergene O Dogan A Gedikli O Hoscan Y. Time interval from the initiation of the electrocardiographic P wave to the start of left atrial appendage ejection flow: a novel method for predicting atrial fibrillation recurrence. J Am Soc Echocardiogr. (2002) 15:1479–84. 10.1067/mje.2002.127515 12464915

[25] Wolf PA Abbott RD Kannel WB. Atrial fibrillation as an independent risk factor for stroke: the Framingham study. Stroke. (1991) 22:983–8. 10.1161/01.STR.22.8.983 1866765

[26] Sanna T Diener HC Passman RS Di Lazzaro V Bernstein RA Morillo CA. Cryptogenic stroke and underlying atrial fibrillation. N Engl J Med. (2014) 370:2478–86. 10.1056/NEJMoa1313600 24963567

[27] Malik S Hicks WJ Schultz L Penstone P Gardner-Gray J Katramados AM. Development of a scoring system for atrial fibrillation in acute stroke and transient ischemic attack patients: the LADS scoring system. J Neurol Sci. (2011) 301:27–30. 10.1016/j.jns.2010.11.011 21130468

[28] Waldenhjort D Sobocinski Doliwa P Alam M Frykman-Kull V Engdahl J Rosenqvist M. Echocardiographic measures of atrial function may predict atrial fibrillation in stroke patients. Scand Cardiovasc J. (2016) 50:236–42. 10.1080/14017431.2016.1175657 27192631

[29] Anaissie J Monlezun D Seelochan A Siegler JE Chavez-Keatts M Tiu J. Left atrial enlargement on transthoracic echocardiography predicts left atrial thrombus on transesophageal echocardiography in ischemic stroke patients. BioMed Res Int. (2016) 2016:7194676. 10.1155/2016/7194676 27822477 PMC5086361

[30] Piotrowski G Banach M Gerdts E Mikhailidis DP Hannam S Gawor R. Left atrial size in hypertension and stroke. J Hypertens. (2011) 29:1988–93. 10.1097/HJH.0b013e32834a98db 21881527

